# Overexpression of SSAT by DENSPM treatment induces cell detachment and apoptosis in glioblastoma

**DOI:** 10.3892/or.2011.1592

**Published:** 2011-12-14

**Authors:** YE TIAN, SHIZHAO WANG, BIN WANG, JIANNING ZHANG, RONGCAI JIANG, WEI ZHANG

**Affiliations:** 1 Department of Neurosurgery, Tianjin Medical University General Hospital, Tianjin Neurological Institute, Key Laboratory of Post-trauma Neuro-repair and Regeneration in Central Nervous System, Ministry of Education, Tianjin Key Laboratory of Injuries, Variations and Regeneration of Nervous System, Tianjin; 2 Tianjin Medical University, Tianjin, P.R. China; 3 Department of Pathology, The University of Texas, MD Anderson Cancer Center, Houston, TX, USA

**Keywords:** N^1^,N^11^-diethylnorspermine, spermidine, spermine N^1^-acetyltransferase, glioblastoma, cell detachment, apoptosis

## Abstract

N^1^,N^11^-diethylnorspermine (DENSPM), a polyamine analog that induces expression of spermidine/spermine N^1^-acetyltransferase (SSAT) and reduces polyamine levels in eukaryotic cells, has demonstrated anticancer effects in many cancer cell types. Gene expression of SSAT after treatment with DENSPM was measured in both U87 and LN229 cells using real-time PCR. Induction of SSAT mRNA using DENSPM resulted in significantly higher levels in U87 cells than in LN229 cells. Furthermore, DENSPM caused marked cell detachment in U87 cells and to a lesser extent in LN229 cells. We hypothesized that elevated SSAT expression plays a key role in DENSPM-induced cell detachment in glioblastoma cells. To investigate whether forced expression of SSAT would lead to reduced cell adhesion and increased cell detachment, we transfected a PCMV-SSAT plasmid into LN229 cells and observed significant cell detachment. In addition, we treated U87 cells with SSAT siRNA together with DENSPM to blunt the induction of SSAT by DENSPM. This resulted in an inhibition of cell detachment in U87 cells compared with the DENSPM treatment alone. Increased SSAT expression by transfection enhanced the DENSPM cell-kill effect in LN229 cells whereas reduction of SSAT by siRNA attenuated the DENSPM cell-kill effect. The protein levels of AKT, mTOR and integrin α5β1, which are members of the cell adhesion and anti-apoptotic signal transduction pathways, were decreased in the PCMV-SSAT transfected LN229 cells. Collectively, these results demonstrate that SSAT induction at least partially plays a role in cell detachment and apoptosis of glioblastoma cells by DENSPM treatment.

## Introduction

Polyamines are essential for eukaryotic cell survival although an increase of polyamines has been shown to be associated with survival of cancers. This indicates that the depletion of polyamines may be a potential strategy for cancer treatment. It has long been known that the depletion of polyamines can induce apoptosis in several tumor types using either a polyamine synthesis inhibitor or a polyamine analog (1,2). However, polyamine synthesis inhibitors have only demonstrated a moderate inhibitory effect on glioblastomas (3).

N^1^,N^11^-diethylnorspermine (DENSPM), a well-studied polyamine analog, was shown in our previous study to induce significant apoptosis in several glioblastoma cell lines (3). Although the mechanism of polyamine analog-induced cell death is still not well understood, it has been shown that DENSPM induces spermidine/spermine acetyltransferase (SSAT) expression, which converts spermine and spermidine to their acetylated forms. Acetylated spermine and spermidine can then serve as substrates for spermine oxidase (SMO) or polyamine oxidase (PAO) in reactions that produce H_2_O_2_ (4,5). In most experiments, DENSPM was described to induce apoptosis in cancer cells by damaging mitochondria, releasing cytochrome c into cytoplasm and activating the caspase-3 and -8 signal transduction pathways (6,7). We have also observed mTOR relocation in U87 cell lines treated by DENSPM (8).

Unexpectedly, significant cell detachment was observed in some glioblastoma cells but not in others. Consistent with our results, other groups have also reported that marked cell detachment could be induced either in CHENSPM (N1-ethyl-N11-[(cyclopropyl)methyl]-4,8,-diazaundecane, another polyamine analog) treated lung cancer cells (9) or in DENSPM-treated breast cancer cells (10). Furthermore, the correlation between increased SSAT levels and cell floating in kidney carcinoma cells (HEK293) was also associated with morphological change in cell shape (11). Based on these findings we hypothesized that elevated SSAT expression plays a key role in DENSPM-induced cell detachment in glioblastoma cells.

## Materials and methods

### Cells and cell culture

The human glioblastoma cell lines LN229 and U87 were purchased from American Type Culture Collection (Manassas, VA) and maintained in Dulbecco’s modified Eagle’s medium (DMEM)/F12 (Gibco, USA) supplemented with 10% dialyzed serum (Hyclone, Logan, UT) at 37°C in a 5% CO_2_ incubator. Because fetal bovine serum has abundant thymine and polyamines, we used the dialyzed serum to avoid interfering with the experimental results.

### Reagents

DENSPM was purchased from Tocris (Ellisville, MO) and was dissolved in water according to the manufacturer’s instructions. The PCMV-SSAT and PCMV empty plasmids (negative control) were kindly provided by Professor Eugene Gerner (Arizona Cancer Center, University of Arizona, Tucson, AZ) and were sequenced in our laboratory before using in order to confirm their quality and their human source.

### In vitro drug treatment experiments

For the *in vitro* drug treatment experiments, the cells were seeded in a 10-cm^2^ dish (10^5^ cells/dish) in 10 ml of medium supplemented with 10% dialyzed fetal bovine serum. Twenty-four hours later, 10 μM DENSPM was added.

### PCMV-SSAT transfection

After LN229 cells reached 80% confluency in the culture plates, they were collected by trypsinization and counted. The PCMV-SSAT and negative control PCMV empty plasmids were transfected into 1×10^6^ LN229 cells in parallel with Nucleofector technology according to the manufacturer’s protocol (Amaxa Biosystems, Gaithersburg, MD). Before transfection, a GFP plasmid was added into the target plasmids to serve as the illumination marker to confirm successful transfection. The transfected LN229 cells were distributed into 10-cm^2^ dishes and continuously cultured in the humidified incubator containing 5% CO_2_ at 37°C for another 24 h.

### Knockdown of SSAT expression by siRNA

Dharmacon SMARTpool^®^ siRNAs (Dharmacon, Lafayette, CO) were used for silencing SSAT with Nucleofector technology according to the manufacturer’s protocol. For the non-specific target, nonsense siRNA (Ambion Inc., Austin, TX) was used as a control. Briefly, 2–3×10^6^ LN229 or U87 cells were resuspended in 100 μl of Nucleofector solution with 100 nM of siRNA in the electroporation cuvette. After electroporation, cells were divided into 12-well plates and incubated in the transfection reagent with siRNA at 37°C in a humidified incubator with 5% CO_2_ for 24 h. Following the transfection procedure 10 μM DENSPM was added into the plates.

### Real-time quantitative PCR analysis

The total RNA was extracted using TRIzol (Invitrogen, USA) according to the manufacturer’s protocol. The mRNA level of SSAT from the PCMV-SSAT or PCMV empty plasmid transfected LN229 cells, SSAT siRNA- or nonsense siRNA-transfected U87 cells, DENSPM-treated and untreated U87 and LN229 cells were quantified using the Applied Biosystems TaqMan method in conjunction with Assays-On-Demand (ABI Prism 7900 sequence detection system, Applied Biosystems, Foster City, CA) based on the previous description (6). The results of real-time PCR were analyzed by the ΔΔCT method: ΔCT = CT_selected gene_ - CT_GAPDH_, ΔΔCT = ΔCT_therapy group_ - ΔCT_control group_, RV (relative value)_therapy group_ = 2^−ΔΔCT^, RV_control group_ = 1. The results of real-time PCR were presented as the ratio between the selected genes and GAPDH transcripts. The mean value of SSAT was calculated based on triplicate experiments.

### Cell detachment examination

To evaluate the detachment status of cells treated with 10 μM DENSPM or after PCMV-SSAT transfection or knockdown of SSAT, floating cells in the medium were collected first and then the adherent cells were collected by trypsinization. The percentage of detached cells was calculated by dividing the amount of the total floating cells and the trypsinized adherent cells by the number of the floating cells in the medium. The mean percentage of the detached cells was calculated based on triplicate experiments.

### Cell viability assay

Cell viability was evaluated using the MTS assay (Promega Corporation, Madison, WI). For the MTS assay, we seeded 3,000 LN229 cells transfected with PCMV-SSAT or transfected SSAT siRNA per well in 100 μl of medium in a 96-well plate. On the second day, varying concentrations of DENSPM were added to the wells. After 20 μl of MTS solution had been added to each well and mixed, the cells were incubated at 37°C in the 5% CO_2_ incubator. Absorbance at 490 nm was measured with a microplate reader (MRX, Danatech Laboratory, Houston, TX). All MTS assays were performed in triplicate for each treatment condition and experiments were repeated at least twice to confirm the consistency of results.

### Western blotting

LN229 cells transfected with PCMV-SSAT were lysed and homogenates were clarified by centrifugation at 12,000 × g for 15 min at 4°C. Supernatant samples were electrophoresed on 7.5% sodium dodecyl sulfate (SDS)-polyacrylamide gels followed by transfer to polyvinylidene difluoride (PVDF) membranes (Millipore, USA). Incubation with primary polyclonal antibodies against integrin α5 (BD Biosciences, San Jose, CA), integrin β1 (BD Biosciences), AKT, mTOR (Cell Signaling Technology, Danvers, MA), SSAT (Santa Cruz Biotechnology, Santa Cruz, CA) and actin (Sigma, St. Louis, MO) were performed at a dilution of 1:1,000 overnight at 4°C. After washing, the membranes were incubated with secondary antibodies conjugated to biotin (Amersham Pharmacia Biotech, Piscataway, NJ) at a dilution of 1:3,000 for 30 min at room temperature. Reactions were developed with ECL or ECL plus (GE Healthcare, Buckinghamshire, UK). All Western blotting assays were performed in triplicate for each probed protein.

### Statistical analysis

The SPSS16.0 software (SPSS, Chicago, IL) was used for statistical analysis. Differences in means were analyzed by the two-tailed t-test, assuming unequal variances. All data are expressed as mean ± SD. A P-value <0.05 was considered significant.

## Results

### Cell detachment in DENSPM-treated glioblastoma cells is dependent on SSAT expression levels

**Figure 1:**
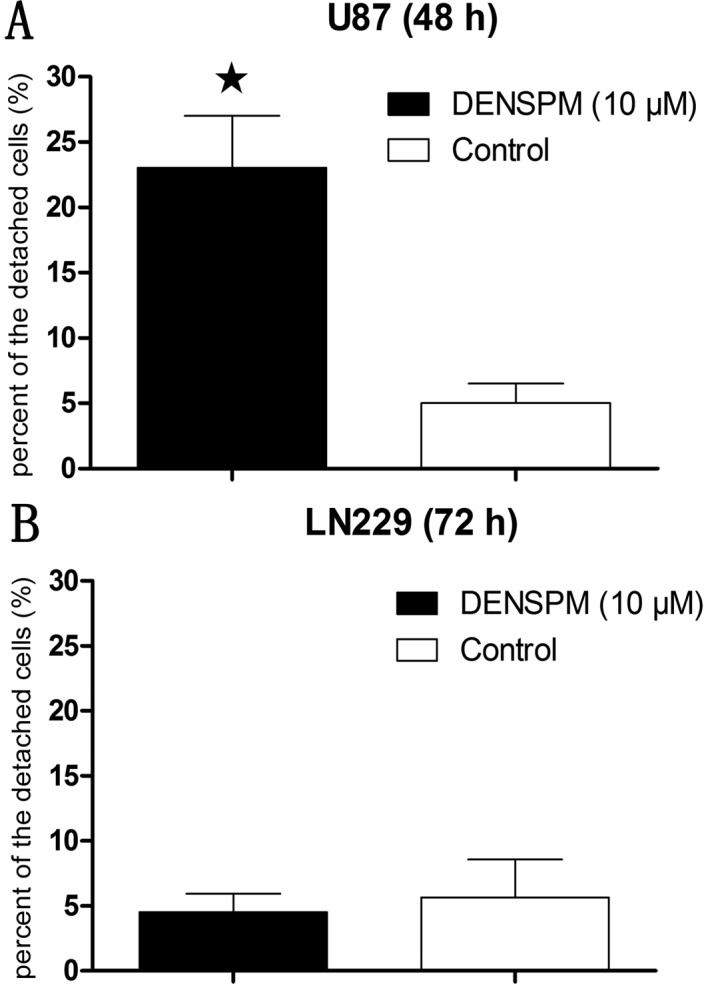
Cell detachment in both U87 and LN229 cells after treatment with DENSPM. (A) The percentage of floating U87 cells increased after incubation with DENSPM for 48 h relative to the control group (^★^P<0.05). (B) Cell detachment was not significant in LN229 cells treated with DENSPM for 72 h.

**Figure 2:**
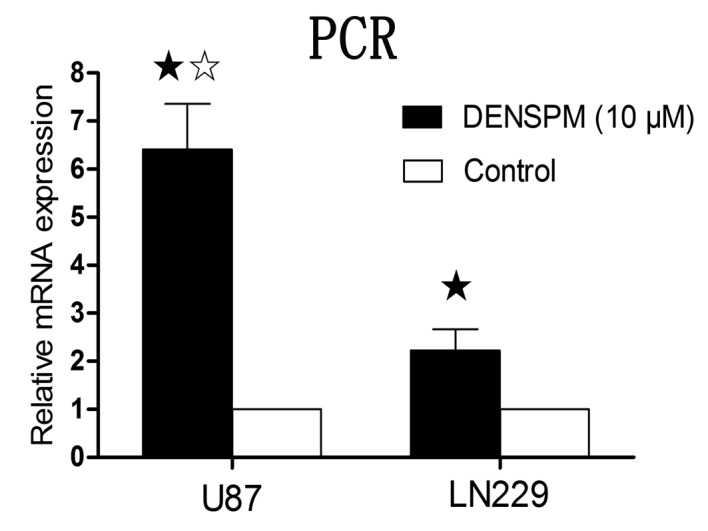
Expression of SSAT mRNA was higher in both U87 and LN229 cells after adding DENSPM compared with control groups (^★^P<0.05). The effect was significantly greater in U87 than LN229 cells (^⋆^P<0.05).

After incubation with DENSPM (10 μM) for 48 h, the percentage of floating cells in U87 cells was ~25% (Fig. 1A). While the incubation time of the same concentration of DENSPM in LN229 cells was extended up to 72 h, this did not result in any significant cell detachment relative to control (Fig. 1B). The measurement of SSAT mRNA level with the real-time quantitative PCR revealed that 10 μM of DENSPM induced significantly increased expression of SSAT in both U87 and LN229 cells. The induction of SSAT mRNA level was significantly higher in U87 cells than in LN229 when incubated for the same period of 24 h (Fig. 2).

### Overexpression of SSAT in LN229 cells induced by transfection of the PCMV-SSAT results in increased cell detachment

**Figure 3:**
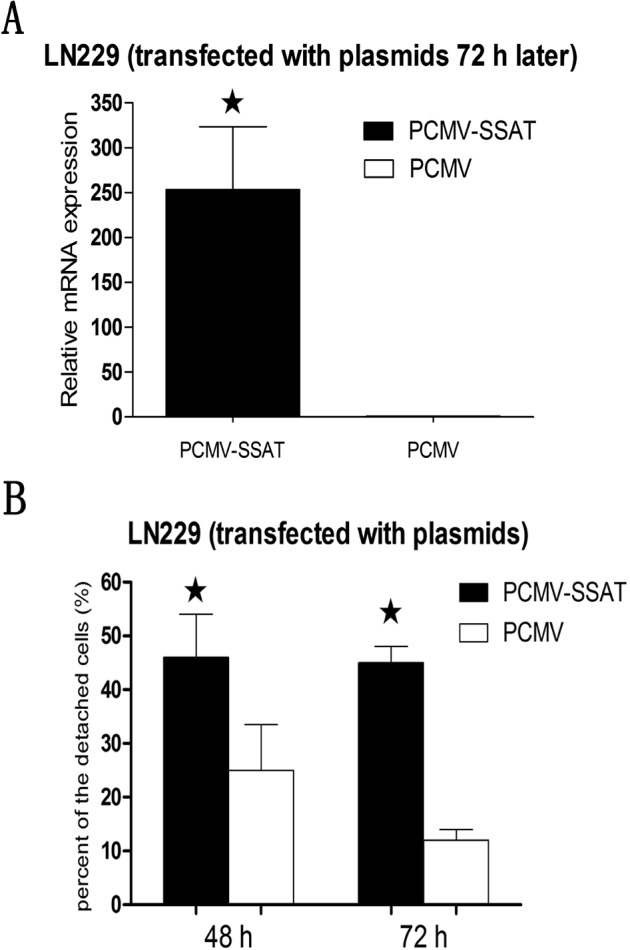
Overexpression of SSAT in LN229 cells transfected with PCMV-SSAT results in greater cell detachment. (A) SSAT mRNA was significantly elevated in PCMV-SSAT-LN229 cells after 72 h compared with PCMV-LN229 controls (^★^P<0.05). (B) The percentage of floating cells in PCMV-SSAT-LN229 cells increased significantly after 48 h and 72 h compared with the PCMV-LN229 control group (^★^P<0.05). There was no significant difference between 48 and 72 h within PCMV-SSAT transfected cells.

The plasmids of PCMV-SSAT were successfully transfected into LN229 cells. Real-time quantitative PCR was performed to confirm that SSAT mRNA was increased in the PCMV-SSAT-LN229 cells after 72 h (Fig. 3A). Then the percentage of the floating cells in the PCMV-SSAT-LN229 cells increased significantly after both 48 h and 72 h compared with cells transfected by PCMV empty plasmids. There was no significant difference in SSAT levels between 48 h and 72 h in transfected cells (Fig. 3B).

### SSAT knockdown inhibits cell detachment in DENSPM-treated U87 cells

**Figure 4:**
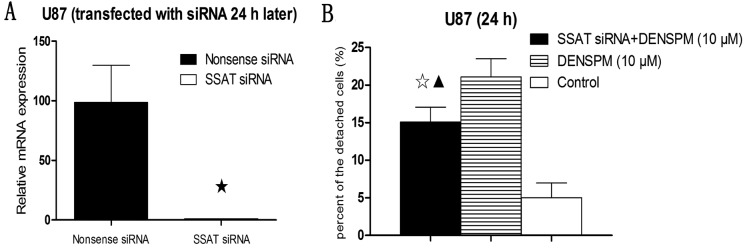
Knockdown of SSAT inhibited cell detachment in DENSPM-treated U87 cells. (A) SSAT mRNA was significantly reduced in SSAT siRNA transfected U87 cells after 24 h compared with the nonsense siRNA transfection group (^★^P<0.05). (B) The percentage of the floating cells in SSAT siRNA transfected U87 cells treated with DENSPM decreased significantly compared with DENSPM treatment alone (^⋆^P<0.05). However, the degree of cell detachment was still significantly higher compared to control group levels (^▴^P<0.05).

Knockdown of SSAT expression with siRNA was confirmed with real-time quantitative PCR (Fig. 4A). Detached cells were counted in DENSPM-treated and untreated U87 cells. A significant reduction in cell detachment was observed in SSAT siRNA-transfected U87 cells treated with DENSPM compared with the DENSPM alone treatment but was still significantly higher than in the control group (Fig. 4B).

### Levels of SSAT affect DENSPM-mediated cytotoxicity in LN229 cells

**Figure 5:**
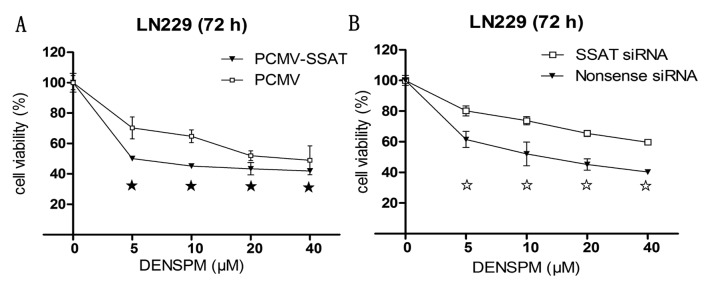
The level of SSAT expression was positively associated with DENSPM’s cytotoxic effects in LN229 cell lines. (A) Cell viability was decreased in LN229 cells following induced SSAT mRNA expression (^★^P<0.05). (B) Conversely, cell viability was higher in SSAT siRNA-treated cells compared with the nonsense siRNA group (^⋆^P<0.05).

Following successful SSAT upregulation by PCMV-SSAT transfection or knockdown by SSAT siRNA, an MTS assay was performed to determine the cytotoxicity of DENSPM in the LN229 cells with serial concentrations of DENSPM. An elevation of SSAT resulted in enhanced cell killing in DENSPM-treated PCMV-SSAT-LN229 cells (Fig. 5A), while a mild attenuation of the DENSPM killing result was observed after the SSAT was turned down (Fig. 5B).

### SSAT transfection induces degradation of anti-apoptosis and adhesion-related proteins

**Figure 6:**
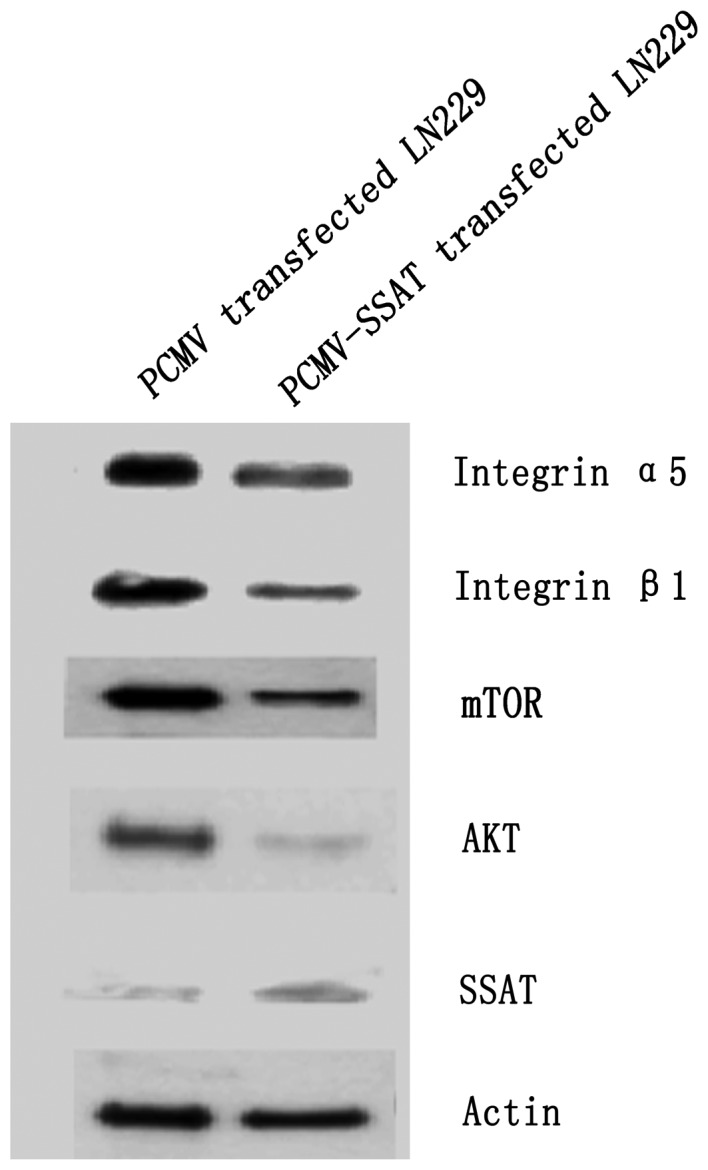
Reduced expression of anti-apoptosis related proteins AKT, mTOR and the adhesion-related proteins integrin α5, integrin β1 in PCMV-SSAT-LN229 cells.

PCMV-SSAT was transfected in LN229 cells and confirmed by the real-time quantitative PCR. Expression of SSAT protein was found to be increased by 256.52% after transfection. The cell lysates were extracted after 48 h and Western blotting run for expression of anti-apoptosis-related proteins AKT, mTOR and the adhesion related proteins integrin α5, integrin β1. SSAT transfection reduced AKT, mTOR, integrin α5 and integrin β1 expression by 35.90, 77.06, 79.91 and 47.33%, respectively (Fig. 6).

## Discussion

Polyamines are essential requirements for eukaryotic cell growth. The metabolism of polyamines are frequently dysregulated in cancer, and the polyamine pathway is a main target for inhibiting the proliferation of carcinoma (2). Polyamine analogues resulting in polyamine depletion in cells are highly attractive chemotherapy targets for cancer. Polyamine analogues can enter into cells, compete with the natural polyamines for uptake but do not substitute for the natural polyamines in growth-related functions. This translates to polyamine analogue uptake in cancer cells resulting in inhibition of polyamine biosynthesis and polyamine catabolism (12). This is a key reason why polyamine analogues are more effective in cancer treatment than inhibitors of the polyamine biosynthesis enzymes. Significant induction of apoptosis by DENSPM was shown to occur in glioblastoma cell lines in our previous report (3). A preliminary exploration on the apoptosis-related signals induced by the polyamine analogue, DENSPM, has revealed that the AKT and the mTOR pathway play an important role in this process. SSAT expression has been noted to be induced after DENSPM application. However, its role in the apoptosis induction by a polyamine analog is far from clear (3,8).

We first demonstrated that the rapid and significant increase in SSAT mRNA was associated with marked cell detachment and cell death in 2 glioblastoma cell lines. Porter and Casero have reported that the cell growth inhibition was caused by the excess induction of SSAT (13,14). The SSAT activity in response to polyamine analogues has been shown to increase in many cell types (15,16), but cannot be overexpressed in some cancer cell types (9,17). DENSPM caused marked cell detachment in U87 cells and to a lesser extent in LN229 cells (Fig. 1). The mechanism responsible for the observed results of SSAT in different cell lines is not well understood. Our results support that the specificity of cell types determines the effect of SSAT cell adherence in response to DENSPM.

To evaluate whether elevated SSAT expression plays a key role in DENSPM-induced cell detachment and cell death in glioblastoma cells, the plasmid construct, PCMV-SSAT was developed to regulate the expression of SSAT in the tumor cells. Our results confirmed that the expression of SSAT was involved in determining the degree of the cell detachment and death consistent with findings by other groups. Hegardt and colleagues have reported that the elevation of SSAT activity in DENSPM-treated human breast cancer cells (L56Br-C1) induces whole cell detachment after 48 h incubation and a total cell death 72 h later (10). An overexpression of SSAT in kidney HEK 293 cells was also found to cause morphological shape change, the loss of cell anchorage, and the significant alteration of actin-containing filopodia which strongly suggested that the adhesion defect was associated with the high SSAT expression (11).

To further confirm that the induction of SSAT is associated with the cell adhesion, several adhesion-related proteins were examined. Integrins, control cell migration and proliferation through the interaction between neighboring cells and the surrounding extracellular matrix (ECM). Although several integrins have been recognized as key regulators in glioblastoma growth and angiogenesis (18), antagonism of the nonpeptidic α5β1 integrins has demonstrated anti-proliferative activity in glioblastoma cells and inhibition of cell adhesion *in vitro* and *in vivo*. Antagonism of α5β1 integrins are believed to inhibit angiogenesis impairing adhesion and migration of endothelial cells (19,20). In our study, the expression of integrin α5 and integrin β1 was down-regulated in PCMV-SSAT transfected LN229 cells detected by Western blotting. The low expression of both integrin α5 and integrin β1 following SSAT induction correlates with increased cell detachment.

Increased SSAT expression was associated with apoptosis in the glioblastoma cells as demonstrated by decreased expression of anti-apoptosis-related signaling proteins AKT and mTOR. Both were deactivated in SSAT up-regulated glioblastomas and have been recognized as promising targets for therapeutic interventions (21–23). Moreover, simultaneous blockage of AKT and mTOR has been shown to markedly affect proliferative arrest in xenografted tumors (24). Interestingly, the overexpression of SSAT down-regulated AKT and mTOR expression in glioblastoma cells suggesting an effect on cell survival, since the DENSPM-inducing AKT and mTOR down-regulation has been proven to lead glioma cells to apoptosis (8).

Chen *et al* have reported that SSAT binds to integrin α9 and overexpression of SSAT increases integrin α9β1-mediated migration without decreasing cell viability. Furthermore, siRNA knockdown of SSAT inhibited this migration without affecting cell adhesion (25), which is in contrast to our findings. However, the association of SSAT with α9β1-mediated migration supports the notion of SSAT’s role in cell adhesion and adhesion related apoptosis (26,27).

In conclusion, our findings suggest that SSAT plays a partial role in adhesion related to cancer cell survival as evidenced by the subpopulation of cancer cells that responded to SSAT up-regulation. Further studies are warranted to confirm the function of SSAT in the treatment of glioblastoma.
